# *b* map evaluation and on-fault stress state for the Antakya 2023 earthquakes

**DOI:** 10.1038/s41598-023-50837-3

**Published:** 2024-01-18

**Authors:** V. Convertito, A. Tramelli, C. Godano

**Affiliations:** 1 Istituto Nazionale di Geofisica e Vulcanologia - Sezione di Napoli, Osservatorio Vesuviano, Napoli, Italy; 2 Department of Mathematics and Physics, Università della Campania - Luigi Vanvitelli, Caserta, Italy

**Keywords:** Seismology, Geophysics

## Abstract

The analysis of on-fault seismicity can enlighten the current stress state on the fault itself. Its definition is relevant to individuate fault patches that have not released all the accumulated stress even after the occurrence of a high magnitude earthquake. We use the *b* value to characterize the stress state on the fault of the Antakya 2023 main events, being *b* inversely proportional to the stress. The small magnitude seismicity occurring on the maximum slip fault-patches does not allow the *b* value estimation. This represents a strong indication that the maximum slip zone released most of the stress previously accumulated. Conversely, the lowest *b* values are located at the bends of the faults and close to the nucleation zone suggesting that, there, still exists not released stress implying that it could be reactivated in the future.

## Introduction

The occurrence of large aftershocks can cause significant damage in the hours immediately following strong earthquakes^1^. Consequently, their prediction, in terms of magnitude, space and time distributions, represents a crucial point in the risk mitigation. Several time-dependent forecasting models have been proposed for this purpose. Some^1,2^ are based on the Omori-Utsu law^3,4^, and others on the logarithmic envelope of the seismic signal^5–7^ and both provide a satisfactory answer to the aftershocks prediction.

A different approach involves the evaluation of the stress state of a given seismogenic area. Here we use the *b* value of the Gutenberg-Richter (GR) distribution as a strain-meter^8–11^. The GR distribution^12^ states that earthquake magnitude follows an exponential distribution

1 $$\begin{aligned} \log N(m)=a-bm \end{aligned}$$

where the *b* value represents the scaling parameter of the this distribution, namely, the ratio between small and large earthquakes number. The *b* value has been extensively studied because its estimation represents a crucial point in the evaluation of earthquake occurrence hazard. However, here, we consider its inverse correlation with the stress state^8–11^. In order to characterize the spatial variations of the stress state, many efforts have been dedicated to investigate the spatial variations of the *b* value. In particular, the *b* value has been used to characterize the stress state in seismogenic areas^13–15^ or stress regimes^16^, to individuate asperities on active faults^13,14,17^, to discriminate between the on-fault seismicity and distributed one^18^, to enlighten magmatic chambers in volcanic areas^14,19–21^. In all these cases the *b* value is revealed to be a powerful indicator of the stress state and of the fault geometry complexity^22^. It can, thus, help us to understand the physical processes underlying the rock fracture in different tectonic regimes.

The *b* value spatial variations are generally investigated by mapping its value on a space grid covering the earthquake spatial distribution, which are then included in the cells following different rules (e.g., minimum number of events, maximum distance from the centre of the cell, etc.). This approach produces, in some cases, the overlapping of the cells or, in other cases, some earthquakes in the node^23^ are excluded from the analysis. This may prevent a formally correct statistical comparison between different cells of the grid. Some authors weight each earthquake used for the *b* value estimation on the basis of the distance from the grid node. This method^17^ has been applied to several regions of the world (see among the others^24–27^); however, the method introduces correlations in the grid of the *b* values.

Here, we use an automated method^23^ to produce full independent *b* values and reducing the number of missed earthquakes for the analysis of the on-fault aftershocks triggered by the Antakya 2023 earthquakes (see the Data availability for details on the used catalogue). The method allows a better definition of the *b* value on the fault and, consequently, a good description of the afterslip stress state on the fault. A comparison with the slip map of the mainshocks allows us to paint a scenario of the fracturing process. Indeed, the slip may provide^28^ information on the stress drop induced by the occurrence of the two largest events. Conversely, the *b* value map is useful in characterizing the stress state on the fault system in the afterslip phase and, possibly, to enlighten fault patches that could be reactivated in the next future generating strong aftershocks.

## The Turkish seismicity

The $M_w=7.8$ Kahramanmaras earthquake occurred in southern Turkey on February 6th 2023 01:17:35 UTC. It was followed some hours later (10:24:48 UTC) about 100 km away by the Elbistan, $M_w=$7.5, earthquake. These earthquakes are located in an area affected by the interaction of the Arabian and Anatolian plates. This interaction forms the East Anatolian Fault, an over 500 km long left-lateral strike-slip fault that produced large destructive earthquakes in the past few hundred years. East Anatolian Fault consists of several segments where destructive $M>7$ earthquakes nucleated^29^. Interestingly, during the last century, this fault has produced only one large earthquake (*M* = 6.8), whereas the North Anatolian Fault Zone (extending further north between the Eurasian and the Anatolian plate) hosted a remarkable sequence that activated almost the entire fault. Three $M>$7 involved the East Anatolian Fault between 1114 and 1893 (29/11/1114, $M=$7.8; 28/03/1513, $M=$7.4 and 2/03/1893, $M=$7.1)^30^, while only one large earthquake occurred during the last century (4/12/1905, *M* = 6.8)^31^. The apparent seismic quiescence was interpreted as an indication that the fault is currently locked^32^.

## Results

**Figure 1:**
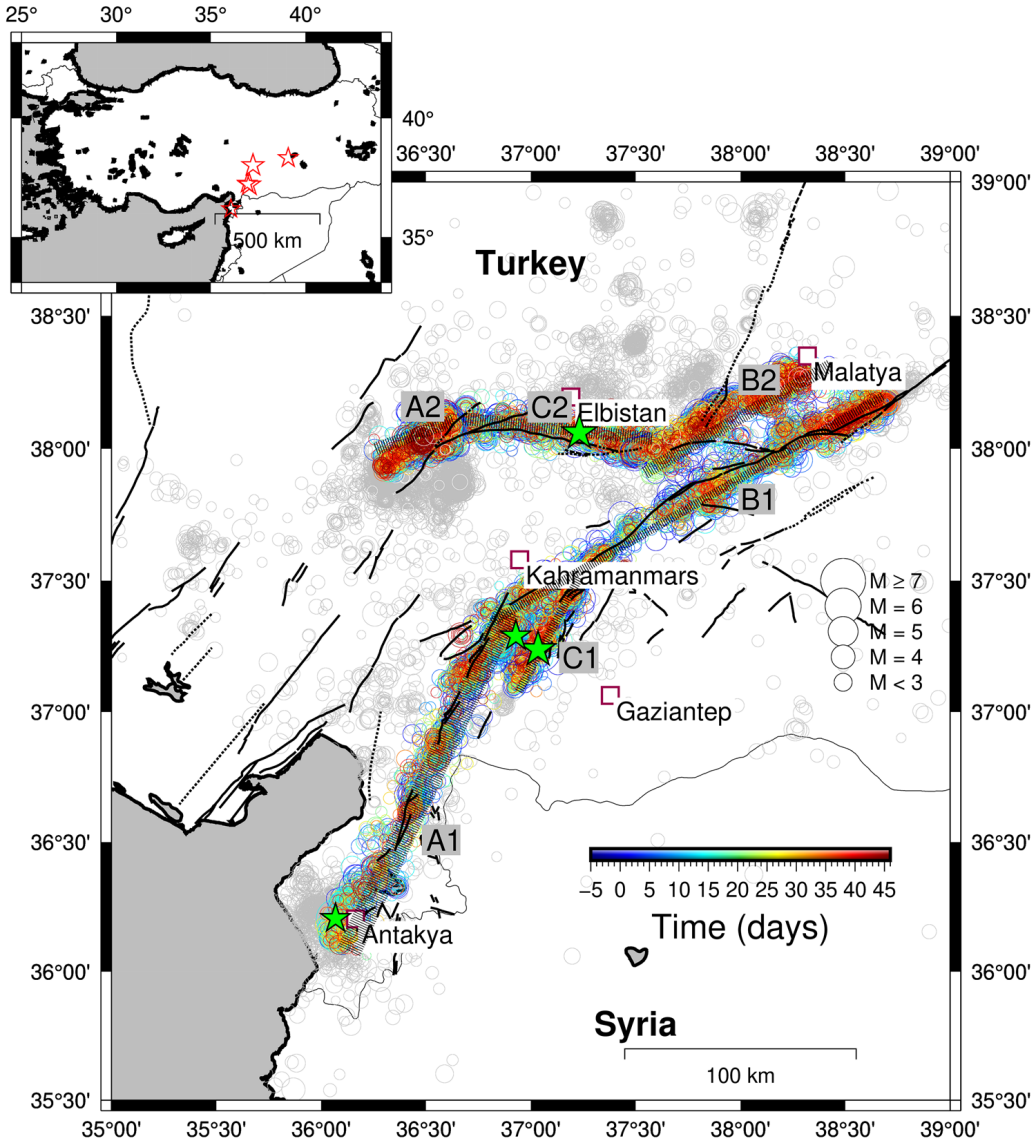
Map of the earthquakes occurred during the analyzed period (see text for detail). Green stars identify earthquakes with magnitude larger than 6. The circle size is proportional to the earthquake magnitude. Grey circles represent all the seismicity included in the catalogue here studied whereas the coloured ones are the on-fault events (see Text for details). The colour code represents the time elapsed since the mainshock. The dark grey lines represent the surface projection of the entire fault used to infer the slip distribution^33^. The map was generated using the program GMT6.4^34^.

In Fig. 1 we show the map of the earthquakes occurred from January 01, 2023 to March 24, 2023 and reported in the catalogue here analyzed. The seismic zone is characterized by a complex fault system. However, the investigated seismicity occurs on two of them, each one composed of three different segments indicated in the figure with different letters. The colour code discriminates between the off-fault earthquakes and the one selected for the on-fault evaluation of the *b* value map. The entire catalogue was composed by 17248 events with magnitude larger than 1.5 and the selection criterion, that is excluding all the off-fault earthquakes (see “Methods” for details), lives 12795 earthquakes in the catalogue. This enlightens that $\approx 75\%$ of the seismicity can be considered as on-fault.

After the application of the method (see the “Methods” section), we were able to evaluate the *b* value in 27 of the 43 unstructured cells selected by the algorithm. This implies that 16 cells were removed from the analysis because of the criteria here applied. More precisely, 8 cells have been discarded because $m_{max}-m_{min}<1.5$ and 8 because $m_{max}-m_c<1.5$. Then we remove, in each cell, all the events with $m<m_c$. The total number of earthquakes left in the catalogue is 4343.

**Figure 2:**
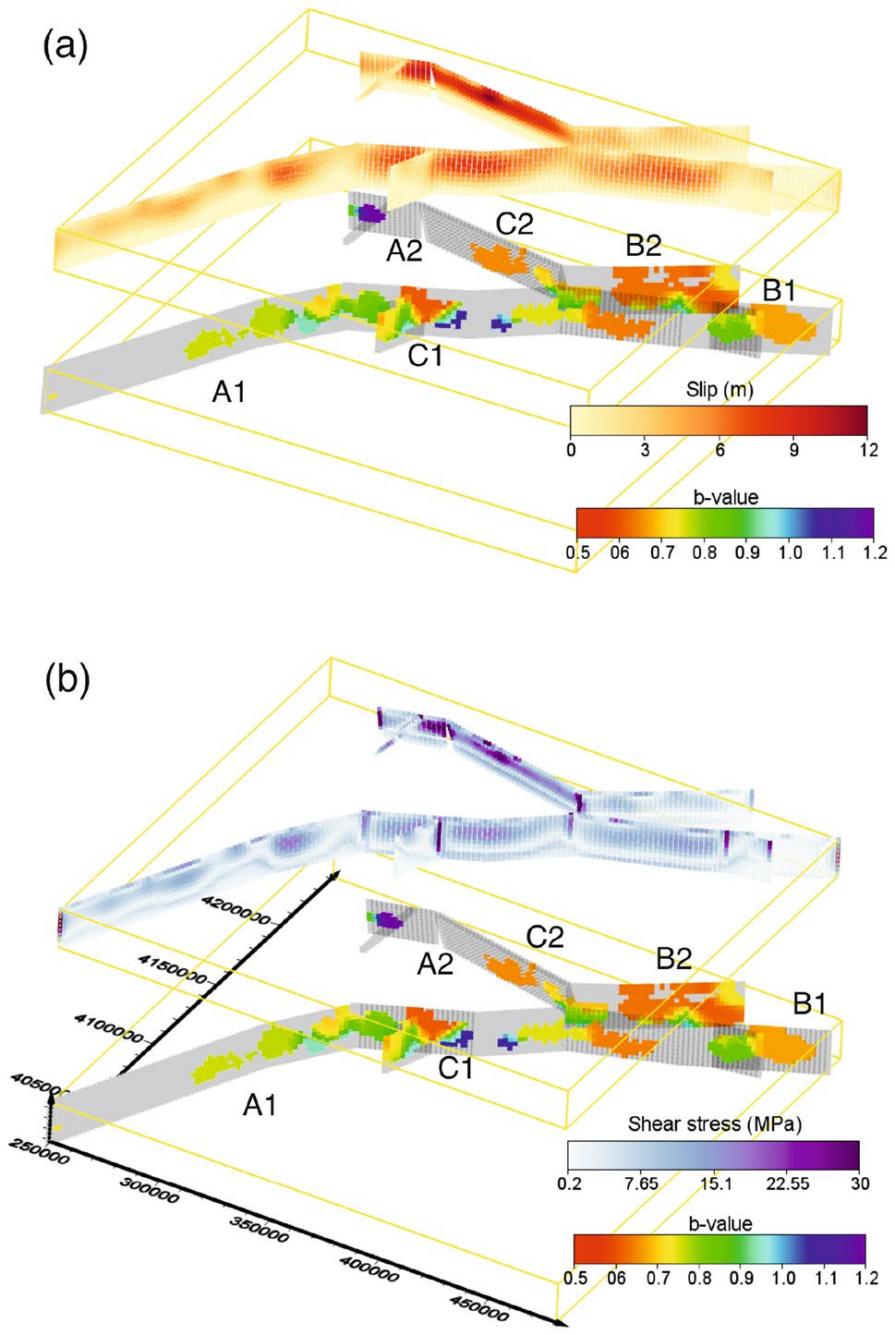
(**a**) Distribution of slip on faults (upper panel) compared with *b* values (lower panel). (**b**) Distribution of shear stress on faults (upper panel) compared with *b* values (lower panel). A movie of the figure, showing the fault for different angles, can be found at the web-site https://zenodo.org/record/7896847. Please do not click on the link, but copy it on the navigation bar. The maps were generated using Voxler Version 3.3.1843 www.goldensoftware.com/.

The final distribution of the *b* value on the faults is shown in Fig. 2 where it is compared with the slip and the shear stress distributions obtained by^33^.

## Discussion and conclusions

The distribution of the *b* value has been both correlated with the stress/strain state in seismogenic volume and with the relative stress distribution on single fault segments^14,35–37^. Moreover, it has been shown that the *b* value distribution is affected by fault geometry in terms of complexity and roughness^38,39^. Here, we compare the *b* value distribution with the slip map of the two main events of the Antakya seismic sequence. We limit to a qualitative comparison because our cells are not assigned to a constant grid whereas slip is evaluated on a constant grid. This makes a point to point association (necessary for a more quantitative correlation analysis) impossible (see Fig. 2). The comparison reveals the following significant characteristics: (1) The large slip zones do not produce aftershocks, making impossible the estimation of the *b* value. This does not exclude a large productivity of mainshocks, but indicates that aftershocks magnitude is lower than $m_c$ or that the magnitude range is limited. (2) Fault segment C1, where the $M_W=$7.8 mainshock nucleated, does not exhibit the largest slip. Here, the *b* assumes values in the range [0.7, 0.8] and we consider such a value as a threshold separating zones with high stress ($b<$0.7) from zones where the stress is smaller ($b>$1.0). (3) Small *b* values, indicating a large stress concentration, are located (a) at the crosses between segments C1 and B1, (b) at the east edges of segments B1 and B2, (c) on areas surrounding the maximum slip patches of segments B2 and C2, (d) at the southern edge of segment C1. (4) More moderately small *b* values (between 0.7 and 0.9) surround the maximum slip zones at the centre of segment B1. (5) Higher values of *b*, indicating small stress, characterize the segment A2 and the deeper seismicity occurring on segment B1. Table 1 reports a summary of *b* values compared with slip and stress.

**Table 1:** Summary of the comparison of slip and stress with the *b* value.

Segment	*b*	Slip (m)	Stress (MPa)
A1	0.7–0.95	0–7	0.2–16
B1	0.65–0.7	<1	0.2–1
C1	0.5–0.9	0–5	0.2–30
A2	0.8–1.2	0–12	0–30
B2	0.6–0.95	0–7	0.2–16
C2	0.65–0.95	0–12	1–30

Very similar considerations can be extrapolated from the shear stress $\tau$ distribution, which was obtained using the^40^ approach, revealing that the higher slip zones are characterized by a larger $\tau$ that is proportional to the stress drop. Again, fault patches with a large $\tau$ exhibit small aftershocks occurrence not allowing the estimation of the *b* value. Conversely, patches with lower $\tau$ are characterized by larger *b* values (see Fig. 2).

These observations suggest a conclusive scenario. The slip of the $M_w$ = 7.8 earthquake, that nucleated on segment C1, was hindered by the presence of segment B1 which, acting as a structural barrier, caused an increasing of the strength at the cross between the B1 and C1 segments. Then, the slip propagated through the B1 and B2 segments. Indeed, it has been observed that the $M_w$ = 7.8 Kahramanmaras earthquake featured a bilateral rupture filling a seismic gap along the East Anatolian Fault Zone^41^. Notably, the eastern edge of the B1 segment is characterized by a small *b* value, indicating a high stress level concentration that precluded, acting as a barrier, rupture propagation^38^. On the other hand, the fracture, propagating through the cross between segments A1 and B1, leaved a not too high stress concentration (*b* values are in the range 0.8–1.0). Maximum slip zones are characterized by the absence of significant aftershocks, implying that the great part of stress was released during fracture propagation. Segment A1 exhibits relatively smaller slip and the absence of aftershocks does not allow the estimate of the *b* value. This is an indication that the fault fracture exhausted its energy without any hindering mechanism. More recently, it has been suggested that aftershock productivity is modulated by fault roughness being the productivity of rough fault higher than the one produced by smooth faults^39^. In this respect, our results suggest that fault segment A1 presents a lower roughness compared with the other segments.

The $M_w$ = 7.5 Elbistan earthquake nucleated on fault segment C2 and slip propagates along this segment and segments A2 and B2. On A2, the slip appears to release the accumulated stress and the seismicity is characterized by higher *b* values. Conversely, the slip appears to be stopped on segment B2 by the presence of a barrier generating a fault zone with a small *b* value. Again, the maximum slip zones appear to have released the great part of the stress and seismicity surrounding the zone of maximum slip is characterized by high levels of stress.

Our results appear to be in excellent agreement with and confirm the ones found for the Cahuilla, California 2016–2019 earthquake swarm^22^ and in laboratory experiments^39^. In fact, they observed that the fault geometry and its roughness control the stress concentration and, consequently, the aftershocks occurrence.

As a concluding remark, we would like to note that the comparison of the slip and the shear stress with the *b* value maps allows the painting of a fracturing scenario, confirming that the *b* value represents a good parameter for the stress state characterization and revealing that, due to the stress concentration, the cross between segments B1 and C1 and the eastern edges of the faults could be reactivated in the future generating strong aftershocks.

## Methods

### Selecting on-fault seismicity

We selected the fault geometry using the model provided by the USGS at the website https://earthquake.usgs.gov/earthquakes/eventpage/us6000jllz/finite-fault for the $M_w$ = 7.8 Kahramanmaraş earthquake and at https://earthquake.usgs.gov/earthquakes/eventpage/us6000jlqa/finite-fault for the $M_w$ = 7.5 Elbistan one. Both the fault segments are 40 km thick, however, we limit to the first 20 km in order to compare the *b* map with the slip and stress ones. We selected, as on-fault seismicity, all the earthquakes occurring within a distance of 10 Km from the fault plane and exclude all the events deeper than the fault thickness. This ensures to be almost outside the location error whose modal values are 1.5 km for the horizontal and 3.1 km for the vertical.

### Building independent cells on faults

As already stated, we used the method described in detail in^23^, to divide the on-fault seismicity into independent cells. The method individuates the largest event in the catalogue not yet assigned to a cell and build, around the chosen earthquake, a cell containing $n\pm n_{tol}$ events. *n* is the only parameter of the method. Here, $n=300$ and $n_{tol}=30$. The method, well described in^23^, does not produce cells of homogeneous geometrical dimensions. In the analyzed catalogue, the cell size varies between 5 and 20 km. However, the three dimensional *b* value is projected on the fault segments following the regular gridding of the slip map to allow the comparison. When $m_{max}-m_{min}<1.5$, the cell is discarded from the analysis. The choice of these numbers appears to be a good compromise in order to have sufficient cells to allow for an interpretation of the observed *b* values. Indeed choosing $m_{max}-m_{min}$ threshold smaller than 1.5 significantly increases the standard deviation of the *b* value, conversely, for $m_{max}-m_{min}$ greater than 1.5, the number of cells respecting the criterion reduces too much and any analysis is not possible anymore. At the same time if $n<300$ the number of cells with $m_{max}-m_{min}\ge 1.5$ reduces significantly as well as if $n>300$.

### Evaluating the completeness magnitude and *b* value

The completeness magnitude $m_c$ is defined as the minimum magnitude over which all earthquakes are reported in the catalogue. Its value is crucial for a correct estimation of the *b* value. Indeed an underestimated $m_c$ leads to an underestimated *b* value. Conversely, an overestimated $m_c$ leads to a reduction in the magnitude interval implying a not correct estimation of the *b* value.

**Figure 3:**
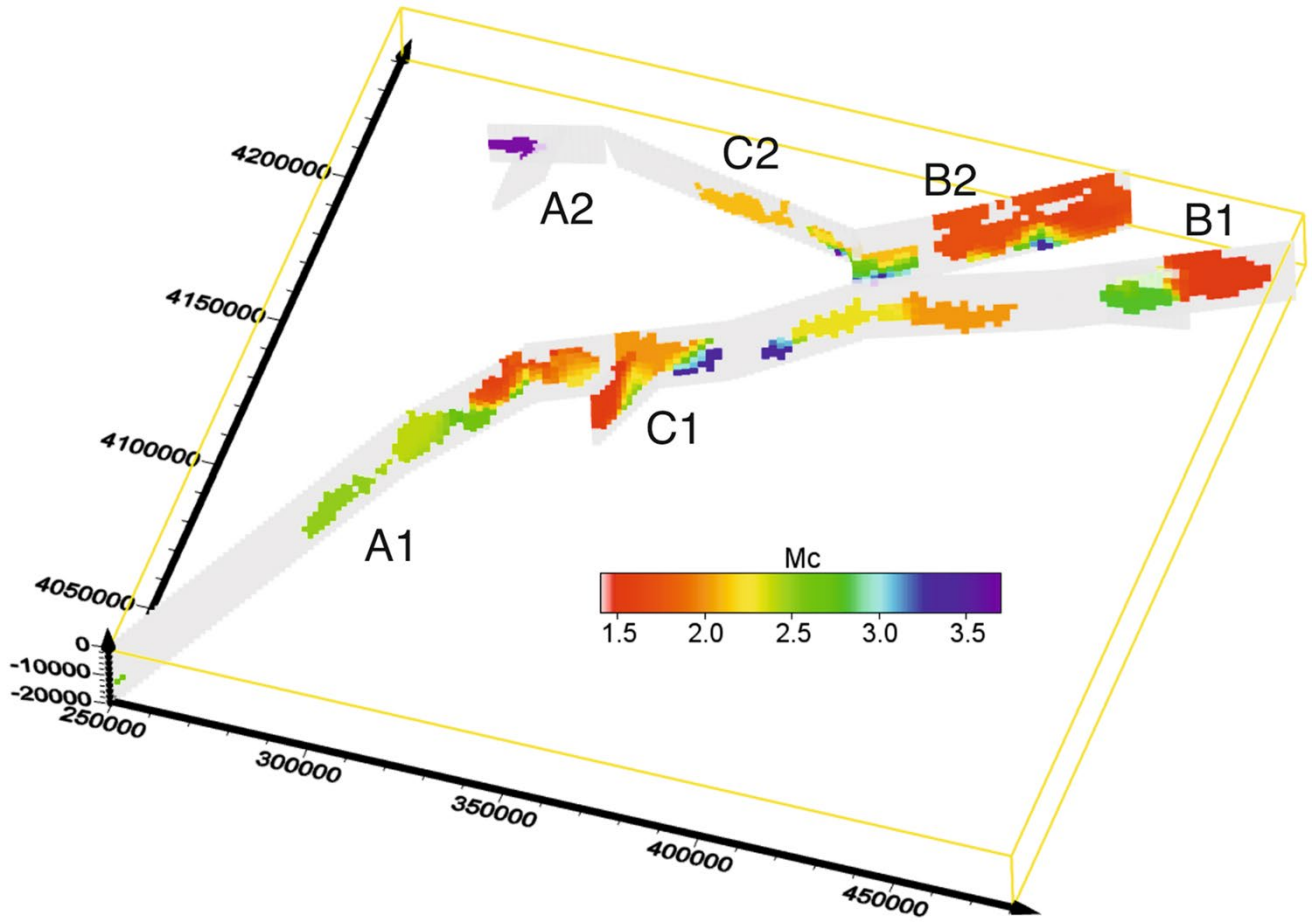
The $m_c$ map. Great part of the cells exhibit a $m_c$ value in the range [1.5,2.2], some of them in the range [2.2,2.8] and very few have $m_c>3$. The map was generated using Voxler Version 3.3.1843 www.goldensoftware.com/.

Following^42,43^ we evaluate the variability coefficient $c_v$ (defined as $\frac{\sigma _{m-m_{th}}}{\langle m-m_{th}\rangle }$ where $\sigma _{m-m_{th}}$ is the standard deviation of $m-m_{th}$) as a function of a threshold magnitude $m_{th}$. When $c_v\simeq 1$ (here we adopt a tolerance of 0.1%), $m_{th}=m_c$. Figure 3 shows a map of the $m_c$ value.

Then we compute the *b* values, only for cells verifying the condition $m_{max}-m_c\ge 1.5$, by using the maximum likelihood method^44^; whereas the estimation of the *b* standard deviations $\sigma _b$ uses the method of^45^. The choice of $m_{max}-m_c<1.5$ comes from the same considerations done for the $m_{max}-m_{min}$ constraint.

An analysis of the *b* value temporal variations in the cells is not possible because the number of events per cell is too small and does not allow it. Conversely, an investigation of the *b* value time variations for the entire catalogue is outside our aims.

## Data Availability

For the present analysis we used the high precision located catalogue obtained due to Smooth Source Specific Station Travel-time Correction and waveform coherence^46^ available at the web-site https://zenodo.org/record/7699882. The on-fault distribution is available at the web-site https://zenodo.org/record/7879743#.ZIbtOXZBxPY.

## Code availability

All the custom codes used in this study are available at the web-site https://github.com/sismanna/b_value_map.
